# Anti-Skin Aging and Cytotoxic Effects of Methanol-Extracted *Solanum betaceum* Red Fruit Seed Extract on Ca9-22 Gingival Carcinoma Cells

**DOI:** 10.3390/plants13162215

**Published:** 2024-08-09

**Authors:** Yen-Hua Huang, Cheng-Yang Huang

**Affiliations:** 1 Department of Biomedical Sciences, Chung Shan Medical University, Taichung City 402, Taiwan; 2 Department of Medical Research, Chung Shan Medical University Hospital, Taichung City 402, Taiwan

**Keywords:** *Solanum betaceum*, anti-skin aging, anticancer, Ca9-22 oral carcinoma, tyrosinase, hyaluronidase, elastase

## Abstract

The tamarillo, or *Solanum betaceum*, recognized for its comprehensive nutritional profile, has long been valued for its diverse ethnobotanical uses. This study delves into the potential therapeutic applications of *S. betaceum* by analyzing its polyphenolic content (TPC), total flavonoid content (TFC), anti-skin aging activities against key enzymes like elastase, tyrosinase, and hyaluronidase, and its cytotoxic effects on oral carcinoma cells. Extracts from the seeds, pulp, and peel of red and yellow fruits were prepared using methanol, ethanol, and acetone. The highest TPC was found in the methanol extract from red fruit seeds (9.89 mg GAE/g), and the highest TFC was found in the methanol extract of yellow fruit peel (3.02 mg QUE/g). Some of these extracts significantly inhibited skin aging-associated enzymes with the red fruit seed extract (100 μg/mL) showing up to 50.4% inhibition of tyrosinase. Additionally, the red fruit seed extract obtained using methanol demonstrated potential anticancer effects against Ca9-22 oral carcinoma cells by inhibiting cell survival, migration, and proliferation as well as inducing apoptosis. These results underscore the potential of *S. betaceum* fruit extracts, especially from red fruit seeds, as promising agents for anti-skin aging and anticancer applications, meriting further exploration for therapeutic uses.

## 1. Introduction

Plants are well recognized as a vast reservoir of pharmaceutical agents possessing therapeutic and prophylactic benefits, which are attributable to their antioxidant, anti-inflammatory, antimicrobial, and antiproliferative properties [1]. A significant benefit of using natural extracts in therapeutic contexts is their capacity for multimodal action [2]. Consequently, exploring the potential of plant extracts for novel therapeutic uses is crucial [3,4,5,6,7], such as investigating the fruit seeds of *Solanum betaceum* in this study for compounds that may prevent skin aging. Skin aging [8] is a multifactorial biological process [9] driven by intrinsic and extrinsic factors that manifest as wrinkles, loss of elasticity, and other visible skin deteriorations [10,11,12,13]. Natural products, particularly those derived from *S. betaceum*, may counteract skin aging by targeting and inhibiting key enzymes responsible for skin matrix degradation [14], including tyrosinase, elastase, and hyaluronidase.

*S. betaceum*, commonly referred to as tamarillo, chilto, or tree tomato [15], is a culinary species celebrated for its distinctive flavor and vibrant color [16]. Native to the tropical and subtropical regions from Colombia to Argentina, this fruit is noted for its comprehensive nutritional profile, including high concentrations of vitamins A, B_6_, C, dietary fiber, and potassium, along with bioactive compounds such as phenolics, carotenoids, and anthocyanins [17]. Recognized for its array of health-promoting properties, *S. betaceum* has been implicated in various therapeutic applications, offering antioxidant, anti-obesity, anticancer, and prebiotic benefits [16]. Consumed primarily in salads, jams, juices, and liquors, *S. betaceum* has also been harnessed by the food industry to create innovative products such as frozen functional pulps, energy drinks, and ice creams [15]. Owing to its superior antioxidant activity, surpassing even that of apples and kiwifruit [16], *S. betaceum* holds significant potential as a functional ingredient in cosmetics and pharmaceuticals. Additionally, this study explores the anti-skin aging capabilities of its constituent parts—pulp, seeds, and peel—through various solvent extractions.

*S. betaceum* contains high amounts of phenolics, which are potent antioxidants capable of inhibiting LDL oxidation in vitro [18]. The HPLC-ESI-MS/MS analysis of phenolic-enriched fractions facilitated the identification of 12 caffeic acid derivatives, 10 rosmarinic acid derivatives, and 7 flavonoids [19]. These polyphenol-rich extracts, both pre- and post-simulated gastroduodenal digestion, demonstrated inhibitory effects on enzymes linked to metabolic syndrome, such as α-glucosidase, amylase, and lipase, and they also showed antioxidant properties [19]. Discovering whether *S. betaceum* possesses other new biological benefits is still worthwhile.

The global prevalence of cancer, particularly oral carcinoma, underscores the imperative need for new therapeutic interventions [20,21,22]. Oral cancer ranks among the top ten most prevalent cancers worldwide, predominantly manifesting in the oral cavity [23]. Oral squamous cell carcinoma (OSCC), the most prevalent form of head and neck cancer, has a concerning five-year survival rate of only 50% [24]. The incidence of OSCC in Asia is notably high, and it is influenced by specific exposure and risk factors. Traditional treatment modalities for oral cancers include surgery, chemotherapy, and radiation therapy [25]. However, these treatments can adversely affect healthy tissues and cells and are often compromised by the development of drug resistance [25]. Consequently, there is a growing interest in leveraging natural compounds as potential anticancer agents alongside conventional treatments [1]. This study investigates the cytotoxic properties of *S. betaceum* seed extract, obtained via methanol extraction, on Ca9-22 gingival carcinoma cells. The seed extract of *S. betaceum*, characterized by a high total phenolic content (TPC), has demonstrated promising preclinical efficacy in inducing apoptosis and inhibiting cell proliferation and migration—key processes in effective cancer management.

Tyrosinase, elastase, and hyaluronidase are enzymes critically involved in the skin aging process, where they contribute to the degradation of the dermal extracellular matrix components. Their overexpression and heightened activity lead to visible aging symptoms that could potentially be mitigated by incorporating specific inhibitors into cosmetic formulations such as hydrogels, creams, or lotions. *S. betaceum*, a fruit known for its nutritional value and minimal side effects when consumed, offers promising bioactive compounds for such applications [15]. This study assesses the anti-skin aging and anticancer potentials of *S. betaceum* fruit extracts. We utilized both yellow (orange) and red varieties of the fruit, extracting bioactive components from their pulp, seeds, and peel using methanol, ethanol, and acetone. Specifically, the seed extract from the red fruit demonstrated a strong inhibitory effect on tyrosinase activity, which is crucial for melanin production control. Overall, the findings from this study underscore the traditional and potential novel therapeutic uses of *S. betaceum* fruits, supporting further scientific investigation into their medical applications.

## 2. Results

### 2.1. Total Phenolic Content (TPC)

Polyphenols [26] are increasingly recognized for their potential as cosmeceuticals or drug candidates based on active confirmation screens [27,28,29,30]. Consequently, we analyzed the total phenolic content (TPC) of *S. betaceum* red and yellow fruits. The fruits were dissected into three components: seeds, pulp, and peel, and each component was extracted using methanol, ethanol, and acetone to determine the TPC. TPC was quantified employing the modified Folin–Ciocalteu method. The TPC values varied, ranging from 0.34 mg GAE/g in the acetone extract of the yellow fruit’s pulp to 9.89 mg GAE/g in the methanol extract from the seeds of the red fruit (Table 1). The highest TPC values were observed in the seed of the red fruit extracted with methanol, followed by the peel of the yellow fruit and the peel of the red fruit, which were both also extracted with methanol.

TPC was quantified using a modified Folin–Ciocalteu method. The absorbance of the developed blue color was measured at 750 nm using a UV/VIS spectrophotometer. Results were compared with standard curves of gallic acid (GAE) and expressed as mg gallic acid equivalent per gram of dry weight (mg GAE/g).

### 2.2. Total Flavonoid Content (TFC)

Flavonoids are natural compounds known for their diverse structure-dependent biological and pharmacological activities [31]. Therefore, we also determined the total flavonoid content (TFC) of *S. betaceum* (Table 2). TFC was quantified using the aluminum chloride colorimetric method. TFC values varied with the lowest being 0.65 mg QUE/g in the acetone extract of the pulp from *S. betaceum* yellow fruit and the highest at 3.02 mg QUE/g in the methanol extract of the peel from *S. betaceum* yellow fruit. The highest TFC values were recorded in the peel of the yellow fruit extracted with methanol, followed by the peel of the red fruit with methanol, and the peel of the yellow fruit with ethanol.

TFC was quantified using the aluminum chloride colorimetric method. Absorbance for both extracts and standard solutions was measured at 510 nm. Results were expressed as milligrams of quercetin (QUE) equivalent per gram of dry weight (mg QUE/g).

### 2.3. Anti-Skin Aging Potential

Skin aging is a universal process that ultimately affects all individuals. In this study, we examined extracts from *S. betaceum* red and yellow fruits for their potential to mitigate skin aging. The evaluation was based on the extracts’ inhibitory effects on key enzymes associated with aging, specifically tyrosinase, elastase, and hyaluronidase. Using known inhibitors as positive controls—epigallocatechin gallate for anti-elastase, kojic acid for anti-tyrosinase, and myricetin for anti-hyaluronidase—the inhibitory activity of the extracts from seeds, pulp, and peel of both fruit types was assessed using various solvents (Table 3). The extracts demonstrated significant anti-aging enzyme inhibition at 100 μg/mL with the highest observed effects being 50.4% inhibition by methanol-extracted red fruit seeds for tyrosinase, 28.1% by ethanol-extracted yellow fruit pulp for elastase, and 20.2% by methanol-extracted yellow fruit seeds for hyaluronidase.

These experiments utilized the following positive controls: epigallocatechin gallate for anti-elastase activity, which showed 48% inhibition at a concentration of 5 μg/mL; kojic acid for anti-tyrosinase activity, demonstrating 69% inhibition at a concentration of 10 μg/mL; and myricetin for hyaluronidase activity, achieving 49% inhibition at a concentration of 10 μg/mL.

### 2.4. Cytotoxic Effects of Methanol-Extracted Red Fruit Seed Extract

**Figure 1:**
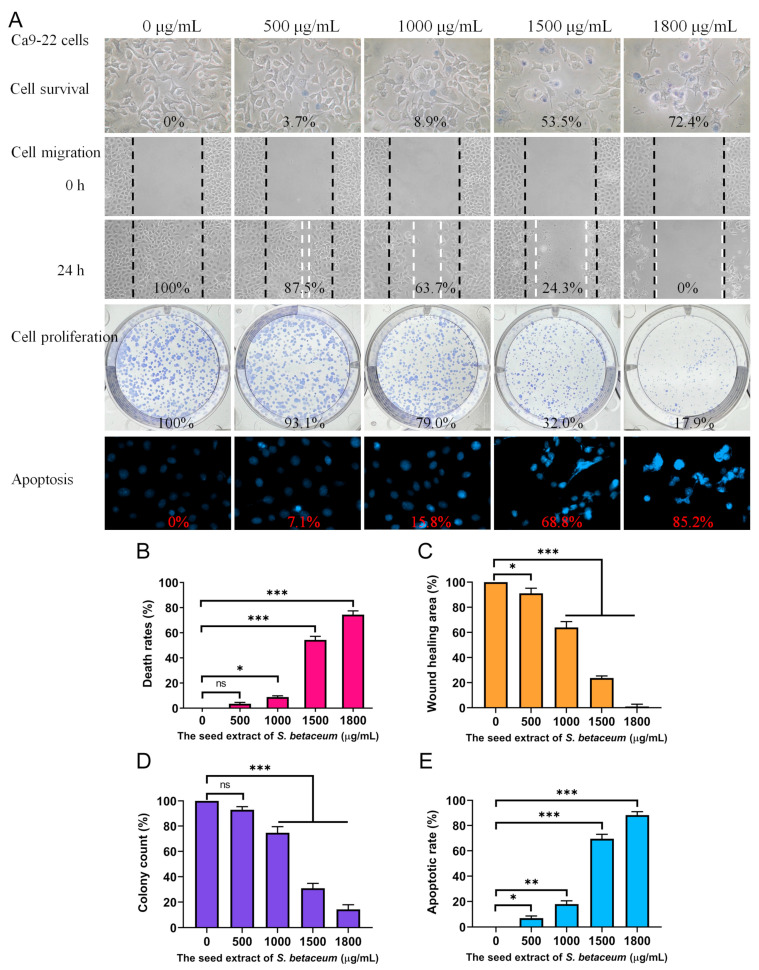
Anticancer potential of methanol-extracted red fruit seeds on Ca9-22 gingival carcinoma cells. (**A**) The impact of the seed extract on cell survival, migration, proliferation, and nuclear condensation in Ca9-22 cells. The black and white dashed lines indicate the positions of cell migration before and after treatment with the extract. (**B**) Trypan blue exclusion assay results demonstrating cell viability after exposure to various concentrations of the seed extract. (**C**) Wound-healing assay depicting Ca9-22 cell migration before and 24 h after treatment at different concentrations of the extract. (**D**) Clonogenic assay assessing the ability of individual cells to form colonies, indicating the survival and proliferative potential of Ca9-22 cells treated with the seed extract. (**E**) Hoechst staining results showing levels of apoptosis and DNA fragmentation at various seed extract concentrations. Statistical significance is denoted by * (*p* < 0.05), ** (*p* < 0.01), and *** (*p* < 0.001) compared to control. ns, not significant.

Cancer continues to be a leading cause of death globally, with mortality rates progressively rising. Polyphenols, abundant in many natural products, are well documented for their anticancer properties. Owing to the high TPC observed in the seeds of the red *S. betaceum* fruit extracted with methanol, this study explored its cytotoxic effects. These effects included the inhibition of cell viability, migration, and proliferation, as well as the induction of DNA fragmentation, which is indicative of apoptosis (Figure 1A). Oral cancer ranks among the top 10 most prevalent cancers worldwide, typically originating in the oral cavity. For this study, Ca9-22 gingival carcinoma cells were employed as the model system. We utilized the trypan blue exclusion assay (Figure 1B) to assess cell viability based on the principle that viable cells exclude the dye due to intact cell membranes, while non-viable cells absorb it, aiding in the differentiation and quantification of live and dead cells. Ca9-22 cells were treated with various concentrations of the extract, which was diluted from a 20 mg/mL stock using culture medium. The cells were incubated with these dilutions or with culture medium containing 1% DMSO as a control. The results revealed that the extract, at concentrations of 500, 1000, 1500, and 1800 μg/mL, resulted in cell death rates of 3.7%, 8.9%, 53.5%, and 72.4%, respectively, suggesting the potential anti-oral cancer efficacy of the *S. betaceum* extract.

### 2.5. Methanol-Extracted Red Fruit Seed Extract Inhibited the Migration of Ca9-22 Cells

The methanol extract from red fruit seeds demonstrated significant anti-migratory effects on Ca9-22 cells, as evaluated using a wound-healing assay (Figure 1C). This assay, a robust in vitro method, assesses collective cell migration across a two-dimensional surface. It involves creating a cell-free gap within a confluent monolayer of cells, which simulates a wound. The cells adjacent to this gap then migrate to close it, mimicking tissue repair. Our observations indicated that treatment with the extract at concentrations of 500, 1000, 1500, and 1800 μg/mL decreased the migration of Ca9-22 cells by 12.5%, 36.3%, 75.7%, and 100%, respectively, within 24 h. These results affirm the extract’s potent inhibitory effect on the migration of oral carcinoma cells, highlighting its potential utility in preventing cancer metastasis.

### 2.6. Methanol-Extracted Red Fruit Seed Extract Inhibited the Proliferation of Ca9-22 Cells

The inhibitory effects of methanol-extracted red fruit seeds on the proliferation of Ca9-22 cells were quantitatively assessed using a clonogenic assay (Figure 1D). This assay evaluates the capacity of single cells to grow into colonies, reflecting their survival and proliferative potential. The results indicated a dose-dependent reduction in both the number and size of colonies formed, signifying a marked impact on cell proliferative capacity. Specifically, treatment with the extract at concentrations of 500, 1000, 1500, and 1800 μg/mL resulted in reductions in colony formation by 6.9%, 21.0%, 68.0%, and 82.1%, respectively. This substantial decrease in colony formation underscores the extract’s potential as an antiproliferative agent, which is capable of reducing the viability and growth of oral carcinoma cells, limiting their potential for progression and spread.

### 2.7. Methanol-Extracted Red Fruit Seed Extract Induced Apoptosis in Ca9-22 Cells

Our results demonstrate that the methanol extract from red fruit seeds effectively induced apoptosis in Ca9-22 cells, as evidenced by Hoechst 33342 staining. This dye, which penetrates all cells, preferentially binds to the condensed chromatin of apoptotic cells, emitting a more intense fluorescence than in non-apoptotic cells. A quantitative analysis of DNA fragmentation, a critical marker of apoptosis, revealed that treatment with the extract at concentrations of 500, 1000, 1500, and 1800 μg/mL led to 7.1%, 15.8%, 68.8%, and 85.2% increases in DNA fragmentation, respectively (Figure 1E). Morphological indicators of apoptosis, including chromatin condensation and nuclear fragmentation, were more pronounced at higher extract concentrations, suggesting a dose-dependent increase in apoptotic cell death. These apoptotic features were distinctly marked by the enhanced blue fluorescence of the Hoechst dye upon binding to apoptotic nuclei. Therefore, the methanol extract of red fruit seeds contains bioactive components that likely activate apoptotic pathways, elucidating the mechanism of action and highlighting its potential therapeutic efficacy against cancer.

## 3. Discussion

In vitro studies have consistently highlighted the health-promoting properties of phytochemicals [32,33]. The skin, as the largest and most complex organ of the human body, serves a critical barrier function against external elements [9]. Its appearance significantly impacts social perceptions, where youthful and healthy skin is often viewed favorably [13]. Aging induces notable changes in skin structure and function, such as increased thinness, dryness, loss of elasticity, rough texture, wrinkle formation, and the appearance of dark spots, which can profoundly influence overall health and quality of life. Therefore, the ongoing identification of anti-aging agents is of great importance [34]. Plant-derived compounds [12], particularly secondary metabolites and whole extracts, have been extensively evaluated for their anti-aging effects [8]. Polyphenols, for instance, are celebrated for their robust antioxidant properties which combat aging and photodamage [35]. Specific polyphenols such as catechins and epigallocatechin gallate are known to target enzymes linked to aging, like elastase [36]. Additionally, natural compounds such as quercetin and myricetin have been identified as inhibitors of key enzymes like tyrosinase and hyaluronidase, which are crucial in the skin aging process [37]. Various quercetin derivatives, including quercetin rhamnoside, rutin, quercetin hexoside, and quercetin glucuronide, have been detected in *S. betaceum* using HPLC–ESI-MS/MS analysis [19]. Investigating whether these quercetin derivatives act as inhibitors against tyrosinase and hyaluronidase merits further exploration beyond the scope of just plant extracts. *S. betaceum*, indigenous to the tropical and subtropical regions from Colombia to Argentina [15], requires abundant sunlight for optimal growth, aligning with the need to explore its potential in mitigating UV-induced photodamage. This study focuses on the anti-skin aging potential of *S. betaceum*, particularly the methanol-extracted red fruit seed extract, which is characterized by high TPC (Table 1) and notable tyrosinase inhibition (Table 3). These findings underscore the importance of investigating specific bioactive compounds and their concentrations in the extract that effectively target key aging-associated enzymes, particularly tyrosinase, to develop effective anti-aging therapies.

Phenolics and flavonoids are widely distributed in plants and constitute the most prevalent polyphenolic phytoconstituents in the human diet. Many phenolics [38] and flavonoids [39] are known to inhibit tyrosinase. We found that significantly high TPC and TFC were found in the methanol extract from red fruit seeds. Therefore, it is evident and unsurprising that *S. betaceum* exhibits anti-tyrosinase activity. It is of considerable interest to further identify which compounds in *S. betaceum* possess strong inhibitory abilities.

The selection of Ca9-22 gingival carcinoma cells was motivated by their direct exposure to compounds in *S. betaceum* during consumption. This model simulates the interaction of these compounds with oral cells, reflecting their dietary intake. While our study offers new insights into the anti-aging and anticancer potential of *S. betaceum* seed extract, we acknowledge its limitations. The research primarily relies on in vitro assays, which are invaluable for preliminary investigations but do not entirely capture the complexity of biological systems. Consequently, future studies employing in vivo models are essential to confirm our results and to evaluate the pharmacokinetics, pharmacodynamics, and potential toxicity of the extract within a physiological context.

Although methanol is less safe compared to ethanol as an extraction solvent, it remains effective for extracting natural products. For instance, using methanol as the solvent for extracting seeds of *Hyoscyamus muticus* has proven significantly beneficial for enhancing TPC and antioxidant capacity [40]. Similarly, this study has identified methanol as the optimal solvent for extracting TPC and TFC from *S. betaceum* red fruit seeds. Methanol’s greater polarity, attributed to its smaller methyl group compared to the larger, more hydrophobic ethyl group in ethanol, results in distinct extraction efficiencies. For future applications of the methanol-extracted *S. betaceum* red fruit seed extract, it is crucial to ensure the complete volatilization of methanol from the extract.

In this study, we determined that the methanol-extracted red fruit seed extract from *S. betaceum* strongly inhibits tyrosinase activity. Tyrosinase, a critical copper-containing enzyme in melanogenesis, catalyzes the hydroxylation of L-tyrosine to L-DOPA and subsequent oxidation to dopaquinone, thus controlling melanin synthesis [41]. Recent research links tyrosinase not only to age spots, photodamage, and skin pigmentation but also to the pathogenesis of Alzheimer’s disease—an irreversible neurological disorder characterized by cognitive decline, memory loss, and behavioral changes [42,43]. Alzheimer’s disease is associated with amyloid-beta (Aβ) deposition, neurofibrillary tangles, oxidative stress, cholinergic deficit, and neuroinflammation [44]. L-DOPA, a by-product of tyrosinase activity, has been implicated in neurotoxicity, inflammatory responses, and an increase in tau protein phosphorylation [45]. Elevated tyrosinase activity, particularly in the substantia nigra, is also linked to neuromelanin synthesis [46]. Given the significant side effects of current Alzheimer’s treatments, targeting tyrosinase inhibition might offer a dual therapeutic strategy not only for skin aging but also for slowing the progression of Alzheimer’s disease. Hence, this study’s findings on the inhibitory effects of *S. betaceum* seed extract on tyrosinase justify further exploration into its use as a source of tyrosinase inhibitors for potential applications in both dermatological and neurological therapeutics.

*S. betaceum* holds considerable commercial value, as it is widely consumed in various forms such as salads, jams, juices, and liquors [15,16]. It has been associated with numerous health benefits, including antioxidant, anti-obesity, anticancer, and prebiotic properties. Being a functional food, *S. betaceum* is generally safe for consumption in large quantities without adverse effects. In this study, the methanol-extracted seed of *S. betaceum* demonstrated cytotoxic effects on Ca9-22 oral carcinoma cells by inhibiting cell migration and proliferation and by inducing apoptosis (Figure 1). The HPLC–ESI-MS/MS analysis has been identified the 31 constituents in *S. betaceum*, including the main compounds caffeoylquinic acid and rosmarinic acid [19]. Future research should explore the potential synergistic and polypharmacological effects of these bioactive compounds, focusing on optimal combinations and concentrations for effective cancer treatment strategies.

## 4. Materials and Methods

### 4.1. Materials

The chemicals used in this study were of analytically pure grade purchased from Sigma-Aldrich (St. Louis, MO, USA). The Ca9-22 gingival carcinoma cell lines were obtained from the Food Industry Research and Development Institute, Hsinchu, Taiwan [47]. Ca9-22 cells were cultured in monolayers using Dulbecco’s Modified Eagle Medium (Gibco^TM^; Thermo Fisher Scientific, Waltham, MA, USA) supplemented with 10% fetal bovine serum (FBS), 100 units/mL penicillin, and 100 μg/mL streptomycin. Cells were incubated at 37 °C in an atmosphere of 95% air and 5% CO_2_.

### 4.2. Plant Materials and Extract Preparations

Fresh *S. betaceum* fruits were sourced in July 2022 from a private farm in Nantou County, Taiwan [48]. The fruits were cleaned to remove impurities and inspected for any damage. Extracts from the seeds, pulp, and peel of both red and yellow fruits were prepared using 100% methanol, ethanol, and acetone. The fruit components were dried, chopped into small pieces, and then ground into a fine powder. For the extraction process, 1 g of the powdered plant material was added to a 250 mL conical flask containing 100 mL of the extracting solvent. This mixture was shaken on an orbital shaker for 5 h. The resulting extract was filtered through a 0.45 μm filter, and the solvent was subsequently evaporated using a hot air circulation oven at 40 °C. The dry extracts were stored at −80 °C until required. The extract powder was reconstituted in 20% DMSO to prepare a stock solution at a concentration of 20 mg/mL. For anticancer assays, this stock solution was diluted with supplemented culture medium to achieve the desired concentrations. Cancer cells were then treated with these extract solutions or with culture medium containing 1% DMSO, serving as the treatment and control groups, respectively.

### 4.3. Determination of TPC

The quantification of TPC was carried out using the modified Folin–Ciocalteu method [49]. The absorbance of the blue color developed was measured at 750 nm by using a UV/VIS spectrophotometer (Hitachi U 3300, Hitachi High-Technologies, Tokyo, Japan) [50]. Gallic acid (GAE) of different concentrations was used as the positive control, and the results of the extracts were compared with the standard curves of GAE and are expressed as mg equivalent/g dry weight of plant. Values show the mean standard deviation of at least three independent experiments.

### 4.4. Determination of TFC

The quantification of TFC was carried out using the aluminum chloride colorimetric method [51]. The absorbance of extracts and standard solutions was measured at 510 nm by using a UV/VIS spectrophotometer (Hitachi U 3300, Hitachi High-Technologies, Tokyo, Japan) [52]. Quercetin (QUE) of different concentrations was used as the positive control, and the results of the extracts were compared with the standard curves of QUE and are expressed as mg of QUE equivalent/g dry weight of plant. Values show mean standard deviation of at least three independent experiments.

### 4.5. Tyrosinase Inhibition

The tyrosinase inhibitory activity was evaluated using a spectrophotometric method based on the oxidation of 3,4-dihydroxy-L-phenylalanine (L-DOPA), employing a modified dopachrome method [53,54]. Extracts were initially solubilized in 20% DMSO and subsequently diluted to the desired concentrations using 0.1 M phosphate buffer (pH 6.8). Each reaction mixture, prepared in a 96-well microtiter plate, consisted of 5 μL of the extract, 115 μL of 0.1 M phosphate buffer, 40 μL of mushroom tyrosinase (200 units/mL), and 40 μL of L-DOPA (2.5 mM). The mixtures were incubated at 37 °C for 30 min. For background absorbance correction, a blank was included for each sample, containing all the reaction components except L-DOPA. The absorbance was measured at 475 nm. Kojic acid served as a positive control, demonstrating expected inhibition, while the negative control contained 10% DMSO instead of the extract. The percentage of tyrosinase inhibition was calculated with the formula: Inhibition % = [(*A*_control_ − *A*_sample_)/*A*_control_] × 100.

### 4.6. Hyaluronidase Inhibition

The inhibition of hyaluronidase activity was quantified using a protocol adapted from the existing literature [55]. Each assay was initiated by pre-incubating 25 μL of the test extract with 3 μL of hyaluronidase from bovine testes type I-S (H3506, Sigma-Aldrich, USA) diluted to 0.4 U/mL in a 20 mM phosphate buffer (pH 7.0), which also contained 77 mM sodium chloride and 0.01% bovine serum albumin (BSA). This mixture was incubated at 37 °C for 10 min. Subsequently, 12 μL of 300 mM phosphate buffer (pH 5.35) was added, which was followed by an additional 10 min incubation at 37 °C. Then, 10 μL of a hyaluronic acid substrate solution (0.03% *w*/*v* in 300 mM phosphate buffer, pH 5.35) was introduced, and the reaction mixture was incubated for 45 min at 37 °C to facilitate the breakdown of hyaluronic acid. The reaction was terminated by the addition of 100 μL of acidic albumin solution (24 mM sodium acetate, 79 mM acetic acid, and 0.1% BSA, pH 3.75) and allowed to stabilize at room temperature for 10 min. Absorbance was then measured at 600 nm. Myricetin was employed as a positive control, and 10% DMSO served as the negative control. The degree of hyaluronidase inhibition was calculated using the following formula: Inhibition % = [(*A*_control_ − *A*_sample_)/*A*_control_] × 100.

### 4.7. Elastase Inhibition

The elastase inhibition assay was performed using a modified protocol from prior research [36]. The reaction was set up in 200 mM Tris-HCl buffer (pH 8.0). Porcine pancreatic elastase was prepared at a concentration of 3.33 mg/mL in the same buffer. The substrate N-Succinyl-Ala-Ala-Ala-*p*-nitroanilide (AAAPVN) was solubilized at 1.6 mM in 200 mM Tris-HCl. The test extracts were pre-incubated with elastase for 15 min to allow for interaction. The complete reaction mixture, totaling 250 μL per well, included the buffer, 0.8 mM AAAPVN, 2 μg/mL elastase, and the test extract. Epigallocatechin gallate was used as a positive control, and 10% DMSO served as the negative control. The absorbance was measured at 405 nm immediately following the addition of the substrate and monitored over a 20 min period in a 96-well plate format. The percentage of elastase inhibition was determined using the following formula: Inhibition % = [(*A*_control_ − *A*_sample_)/*A*_control_] × 100.

### 4.8. Trypan Blue Cytotoxicity Assay

Cell viability was assessed using the trypan blue exclusion assay [56,57] to evaluate the cytotoxic effects of the methanol-extracted red fruit seed extract from *S. betaceum*. Ca9-22 cells were seeded at a density of 1 × 10^4^ cells per well and treated with the seed extract in a total volume of 100 μL for 24 h. The assay was conducted to determine the extent of cell death by differentiating between viable (non-stained) and non-viable (blue-stained) cells using trypan blue dye.

### 4.9. Chromatin Condensation Assay

Apoptosis was evaluated in Ca9-22 cells using Hoechst 33342 staining to visualize nuclear condensation and the fragmentation characteristics of apoptotic cells [58,59]. Cells were seeded at a density of 1 × 10^4^ per well in 96-well plates and allowed to adhere for 16 h. They were then treated with the methanol-extracted seed extract from *S. betaceum* red fruit for 24 h. Following treatment, cells were washed with PBS and stained with 1 μg/mL Hoechst dye in darkness for 10 min. Fluorescence images were captured using an ImageXpress Pico Automated Cell Imaging System (Molecular Devices, CA, USA) equipped with DAPI filter cubes. Image acquisition and analysis were conducted using CellReporterXpress Version 2 software.

### 4.10. Clonogenic Formation Assay

The proliferation inhibition of Ca9-22 cells was assessed through a clonogenic formation assay [60]. Cells were plated at a density of 1 × 10^3^ per well in 6-well plates and allowed to attach overnight. Subsequently, the cells were treated with the methanol-extracted seed extract of *S. betaceum* red fruit for a period of 5 to 7 days. Following treatment, cells were washed with PBS, fixed with methanol, and stained with 0.5% crystal violet for 20 min. The colonies were then counted using a light microscope to evaluate the extent of proliferation inhibition.

### 4.11. Wound-Healing Assay

The migration inhibition of Ca9-22 cells was evaluated using a wound-healing assay, which is a recognized method for assessing collective cell migration in a two-dimensional setup [61]. Initially, cells were cultured in serum-reduced medium for 6 h to minimize proliferation. Subsequently, a cell-free gap was created in a confluent monolayer using a sterile pipette tip. After creating the wound, cells were washed and further incubated in serum-reduced medium. Treatment with the methanol-extracted seed extract of *S. betaceum* red fruit was then administered for 24 h, and the ability of cells to migrate and close the created gap was monitored.

### 4.12. Statistical Analysis

Experiments were conducted in triplicate with results presented as mean ± standard deviation (SD). Statistical significance was determined using GraphPad Prism5 (GraphPad Software Inc., San Diego, CA, USA) with one-way ANOVA employed to assess differences between means.

## 5. Conclusions

In this study, diverse extracts from *S. betaceum*, including those from seeds, pulp, and peel of both red and yellow fruits, were prepared using methanol, ethanol, and acetone. These extracts were analyzed for TPC, TFC, and their inhibitory effects on key anti-skin aging enzymes such as elastase, tyrosinase, and hyaluronidase. Significantly high TPC and TFC were observed in the methanol extract of red fruit seeds, clearly indicating that *S. betaceum* exhibits strong anti-tyrosinase activity. Additionally, the cytotoxic properties of the methanol-extracted seed extract from red fruit were evaluated, focusing on the viability, apoptosis, proliferation, and migration of Ca9-22 oral carcinoma cells, suggesting an anticancer potential. These findings collectively suggest the pharmaceutical potential of *S. betaceum* extracts, warranting further exploration for therapeutic use.

## Figures and Tables

**Table 1:** TPC of *S. betaceum* extracts.

	Methanol	Ethanol	Acetone
Red fruit			
Seeds	9.89 ± 0.22	7.08 ± 0.11	1.92 ± 0.03
Pulp	5.65 ± 0.09	2.96 ± 0.04	0.97 ± 0.02
Peel	7.92 ± 0.13	3.61 ± 0.05	2.92 ± 0.05
Yellow fruit			
Seeds	3.61 ± 0.06	3.60 ± 0.06	1.98 ± 0.04
Pulp	5.09 ± 0.10	3.58 ± 0.09	0.34 ± 0.03
Peel	8.37 ± 0.15	3.42 ± 0.07	2.42 ± 0.05

**Table 2:** TFC of *S. betaceum* extracts.

	Methanol	Ethanol	Acetone
Red fruit			
Seeds	2.05 ± 0.06	1.68 ± 0.04	0.69 ± 0.02
Pulp	1.07 ± 0.02	0.78 ± 0.02	0.79 ± 0.03
Peel	2.85 ± 0.09	1.92 ± 0.08	1.14 ± 0.06
Yellow fruit			
Seeds	1.96 ± 0.04	1.56 ± 0.05	0.73 ± 0.05
Pulp	1.43 ± 0.01	0.94 ± 0.02	0.65 ± 0.04
Peel	3.02 ± 0.10	2.11 ± 0.09	1.58 ± 0.07

**Table 3:** Anti-skin aging potential of *S. betaceum* extracts.

Extract (100 μg/mL)	Inhibition%		
	Tyrosinase	Elastase	Hyaluronidase
Red fruit			
Seeds, methanol	50.4 ± 1.6	0.0 ± 0.0	14.1 ± 0.5
Seeds, ethanol	24.7 ± 1.0	0.0 ± 0.0	7.1 ± 0.2
Seeds, acetone	4.4 ± 0.5	0.0 ± 0.0	4.5 ± 0.3
Pulp, methanol	3.6 ± 0.5	0.0 ± 0.0	6.8 ± 0.4
Pulp, ethanol	3.0 ± 0.2	0.0 ± 0.0	6.6 ± 0.2
Pulp, acetone	1.4 ± 0.1	0.0 ± 0.0	3.4 ± 0.3
Peel, methanol	32.7 ± 1.5	3.2 ± 0.4	9.8 ± 0.6
Peel, ethanol	30.4 ± 0.8	3.6 ± 0.3	9.9 ± 0.8
Peel, acetone	6.1 ± 0.4	1.0 ± 0.2	4.7 ± 0.3
Yellow fruit			
Seeds, methanol	3.2 ± 0.2	8.4 ± 0.9	20.2 ± 0.7
Seeds, ethanol	1.1 ± 0.1	8.7 ± 0.6	17.4 ± 0.5
Seeds, acetone	10.8 ± 0.7	1.5 ± 0.2	1.5 ± 0.1
Pulp, methanol	2.3 ± 0.2	26.4 ± 1.2	13.5 ± 0.5
Pulp, ethanol	2.4 ± 0.3	28.1 ± 0.9	14.7 ± 0.4
Pulp, acetone	4.5 ± 0.5	7.4 ± 0.7	15.0 ± 0.3
Peel, methanol	30.7 ± 1.2	13.4 ± 0.8	14.5 ± 0.4
Peel, ethanol	32.4 ± 1.0	14.5 ± 0.6	13.7 ± 0.3
Peel, acetone	11.5 ± 0.4	5.8 ± 0.3	15.5 ± 0.5

## Data Availability

Data are contained within the article.
